# Clinical Outcomes of Percutaneous Left-Atrial Appendage Occlusion with Conscious Sedation without an Anesthesiologist on Site: Results from a Multicenter Study

**DOI:** 10.3390/medicina59112041

**Published:** 2023-11-20

**Authors:** Matteo Bianco, Andrea Carmelo Visalli, Francesco Tomassini, Carloalberto Biolè, Federico Giacobbe, Cristina Rolfo, Enrico Cerrato, Alfonso Franzè, Greca Zanda, Marco Pavani, Amir Hassan Mousavi, Giulia Gobello, Giulio Piedimonte, Paola Destefanis, Maurizio Lazzero, Sara Palacio Restrepo, Dario Celentani, Alessia Luciano, Emanuele Tizzani, Alessandra Chinaglia, Ferdinando Varbella

**Affiliations:** 1 Cardiology Division, San Luigi Gonzaga University Hospital, 10043 Orbassano, Italyc.biole@sanluigi.piemonte.it (C.B.); giacobbefederico1@gmail.com (F.G.); gobello.giulia@gmail.com (G.G.); paoladestefanis@libero.it (P.D.); alessialuciano76@gmail.com (A.L.);; 2 Interventional Cardiology Unit, San Luigi Gonzaga University Hospital, Orbassano and Infermi Hospital, 10098 Rivoli, Italy; tomascard.tomassini@gmail.com (F.T.); greca.zanda91@gmail.com (G.Z.); giulio.piedimonte@gmail.com (G.P.);; 3 Cardiology Division, Infermi Hospital, 10098 Rivoli, Italy

**Keywords:** left atrial appendage, left atrial appendage occlusion, anesthesia, sedation, transesophageal echocardiography

## Abstract

*Background and Objectives*: Percutaneous left-atrial appendage (LAA) occlusion is an important therapeutic option for preventing cardioembolic stroke in patients with non-valvular atrial fibrillation (AF) at high risk of thromboembolic events and with contraindications for oral anticoagulation (OAC). It is usually performed with transesophageal echocardiography (TOE) guidance under general anesthesia (GA). In this retrospective study, we present a multicenter experience of LAA occlusion performed with conscious sedation (CS) without an anesthesiologist on site. *Materials and Methods*: All the patients on the waiting list for LAA occlusion procedure at Infermi Hospital, Rivoli, and San Luigi Gonzaga University Hospital, Orbassano, from October 2018 to October 2022 were analyzed. All the procedures were performed with a Watchman/FLX LAA closure device under TOE and fluoroscopic guidance without an anesthesiologist on site. CS was performed with a combination of midazolam and fentanyl as needed. *Results*: One-hundred fifteen patients were included (age 76.4 ± 7.6 years, median CHA2DS2Vasc 4.4 ± 1.4). CS was performed using midazolam (mean dose 5.9 ± 2.1 mg), adding fentanyl for thirty-nine (33.9%) patients in case of poor tolerance for the procedure despite midazolam. The acute procedural success rate was 99.1%. We observed seven acute severe complications. No patients needed anesthesiological assistance during the procedure, and no cases of respiratory failure necessitating ventilation were reported. In a follow-up after 10 ± 9 months, one case of stroke (0.9%) and one case (0.9%) of transient ischemic attack (TIA) occurred. *Conclusions*: LAA occlusion performed under CS and without the presence of an anesthesiologist on site appears to be safe and effective. It can be an attractive alternative to general anesthesia (GA), as fewer resources are required.

## 1. Introduction

Atrial fibrillation (AF) is the most common sustained arrhythmia worldwide, and its prevalence progressively increases with age. Patients who suffer from AF have a risk of stroke and cardioembolic complications about five times higher than the general population [1]. ESC guidelines recommend anticoagulation with a class of recommendation IA for patients with AF and elevated thromboembolic risk (CHA2DS2-VASc score ≥ 2 in males and ≥3 in females). Although cardioembolic stroke prevention is of paramount importance, the use of oral anticoagulants (OAC), both warfarin and direct oral anticoagulants (DOAC), can cause severe, and sometime fatal, hemorrhagic events [2,3]. This is particularly true for patients with contraindications for long-term anticoagulation like those with non-modifiable predisposing risk factors for bleeding, in whom left-atrial appendage (LAA) occlusion may be an option [4,5]. Moreover, LAA occlusion is also an important option for patients suffering a stroke while on active treatment with anticoagulant therapy and for those requiring concomitant dual antiplatelet therapy (DAPT) with baseline high-bleeding-risk features.

LAA closure can be challenging due to anatomy, risk connected to trans-septal puncture, the presence of comorbidities, and the potential side effects of general anesthesia. Imaging is fundamental to correctly perform the procedure. The most commonly used imaging modalities are fluoroscopy and transesophageal echocardiography (TOE) [6]. Intracardiac echocardiography (ICE) is an alternative technique that minimizes discomfort for the patient and the need for sedation; however, it is burdened by increased costs, an increased technical challenge due to reduced catheter manipulability, the possible need for the dilation of a previous transseptal puncture or even a second transseptal puncture, and finally limited imaging tools, often including the lack of three-dimensional (3D) imaging [7].

The use of TOE during LAA occlusion is a major source of stress and discomfort for patients; therefore, interventions are often performed under general anesthesia (GA) with orotracheal intubation. GA offers the advantage of airway protection and the complete immobilization of the patient but is associated with higher costs, prolonged hospital stay, and adverse effects.

An intriguing alternative to GA is conscious sedation (CS), provided and managed directly by interventional and imaging cardiologists and not requiring the presence of an anesthesiologist in the catheterization laboratory (cath-lab). This option could lead to an increase in aspiration pneumonias and complications due to agitation of the patient. Data about outcomes and safety using CS rather than GA are limited to a few retrospective studies [8,9,10].

In this multicenter retrospective registry study performed in high-volume centers with highly experienced operators, we present the results of LAA occlusion using CS managed by a “cardiologist only” team without an anesthesiologist on site.

## 2. Materials and Methods

### 2.1. Design of the Study and Data Analysis

This is a retrospective, independent, spontaneous, observational, multicentric study. We consulted the electronic database (Ebit, Esaote, Genova, Italy) of the cardiology department of San Luigi Gonzaga University Hospital and Infermi Hospital concerning information from 27 October 2022 to 8 December 2022 in order to collect data about patients who were listed for LAA occlusion procedure for stroke prevention of non-valvular AF in the period from 1 October 2018 to 1 October 2022. San Luigi Gonzaga University Hospital started the percutaneous LAA occlusion program in February 2022. This study was performed according to the San Luigi Gonzaga University Hospital Review Board guidance and was conducted in accordance with the Declaration of Helsinki and its later amendments. Due to the observational and retrospective nature of this study, patient informed consent was waived, but all the data were anonymized before being collected.

The principal investigators (MB, ACV, and FT) were responsible for the study design, maintenance of the database, data validation, and analyses. The statistical expert (EC) provided support for statistical analysis and reviewed the paper draft. The first, second, and third authors wrote the first draft of the article, and the writing committee made revisions and the decision to submit the article for publication.

For each patient, we collected the following variables in a standardized manner: 1. general baseline characteristics, 2. indications for LAA occlusion, 3. previous history of bleeding, 4. procedural characteristics and medications used, and 5. imaging modality used during the procedures. Moreover, we collected data about the echocardiographic follow-ups of the patients and, when available, about clinical variables at a one-year follow-up visit. Only in case of the absence of a one-year follow-up visit, we performed a remote follow-up with the patient’s general practitioner or conducted a phone call directly to the patient. In case of incomplete clinical or procedural data, patients were excluded from the study. Patients who underwent an LAA occlusion procedure guided by intracardiac echocardiography were excluded from the analysis.

### 2.2. Screening and Selection of Patients

All the patients on the waiting list for an LAA occlusion procedure were called for a screening TOE one or two days before the procedure in order to assess the anatomy of LAA and fossa ovalis to rule out LAA thrombosis and to evaluate contraindications and tolerance to TOE under CS. The standard TOE views for LAA visualization were routinely conducted using angles of 0, 45, 90, and 135°, and evaluation of interatrial septum at 45° and using bicaval views was performed. All patients gave informed consent to undergo TOE. Screening TOEs were performed using sedation with midazolam in addition to lidocaine spray, and the cardiologist performing the exam evaluated contraindications for CS and tolerance of the patients for TOE, with a special focus on achieving adequate sedation and the occurrence of paradoxical reactions to benzodiazepines.

Patients with anatomy suitable for the procedure were then admitted to the cardiology ward for the intervention; in case of poor tolerance or overt contraindications for prolonged CS, either anesthetist assistance or ICE guidance during the procedure was arranged.

All TOE procedures were performed with Philips Epiq 7 echocardiographic machines using X8-2t transesophageal transducers (Philips, Amesterdam, The Netherlands).

### 2.3. LAA Occlusion Procedure

All LAA occlusion procedures were performed at San Luigi Gonzaga University Hospital and in Infermi Hospital cath-labs by expert interventional cardiologists (FT, CR, MP, and EC) and were guided via TOE and fluoroscopy.

**Figure 1:**
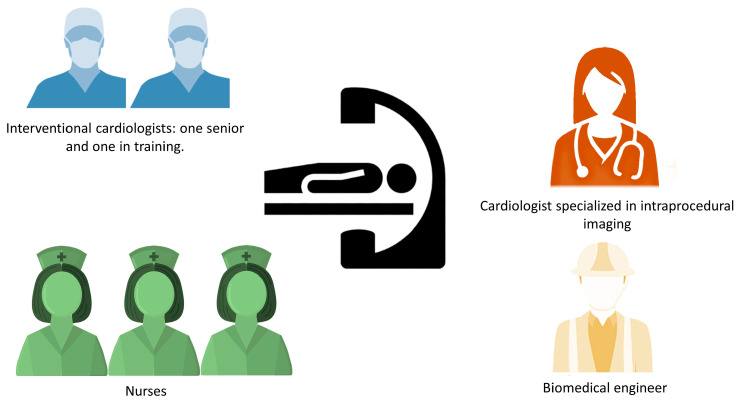
The team present in the cath-lab during LAA occlusion procedure with CS without anesthesiologist on site.

The typical team in the cath-lab during LAA occlusion procedure with CS is described in Figure 1. These teams usually consisted of one expert interventional cardiologist, one interventional cardiologist in training, a cardiologist performing TOE and managing patient’s CS, three nurses (one helping with surgical instrumentation, one administering drugs and monitoring and assisting the patient, and one available for further assistance), and a biomedical engineer aiding in addressing the technical aspects of the procedure.

Venous femoral access site was always secured under echocardiographic guidance. Oropharyngeal anesthesia for the introduction of TOE transducer was induced with lidocaine spray. The standardized protocol for CS started with midazolam bolus dose of 0.05–0.15 mg/kg followed by a continuous infusion of 0.05–0.12 mg/Kg/h on the basis of patient individual response. CS was modulated to achieve sedation, tolerance, and immobility without altering spontaneous breathing. Fentanyl was administered with an intermittent bolus dose of 0.35–0.7 mcg/kg (every 15–30 min) in case of poor tolerance for the procedure and the inability to obtain the desired level of CS. Drug dosages were adjusted on the basis of age and kidney and liver function. Arterial blood pressure, oxygen saturation, and electrocardiographic (ECG) trace were continuously monitored during the procedure.

Transseptal puncture was then performed under echocardiographic guidance in the inferior-posterior part of fossa ovalis. After that, angiographic and TOE measurements of LAA were taken as well as measurements of left-atrial pressure (LAP). After size selection, the device was placed in the LAA and opened, and the position, compression, stability, and sealing of the LAA were checked. In case of suboptimal positioning of the device, recapturing and repositioning attempts were made until the team judged the position to be optimal. Finally, the LAA occluder was definitively released. While the delivery system and the sheath were removed, a check for the presence of pericardial effusion was performed. Then, the TOE probe was removed, and the midazolam infusion was discontinued. In the absence of complications, the patient per protocol returned to the cardiology ward without an observation period in intensive or sub intensive cardiac care units. The patients were subjected to a trans-thoracic echocardiogram 3–6 h after the intervention to re-check for pericardial effusion. ECG monitoring with telemetry was normally maintained until discharge.

During the intervention, unfractionated heparin (UFH) was administered in order to maintain activated clotting time (ACT) of >250 s. Anticoagulant and antiplatelet therapies after closure were managed according to patient bleeding risk as per international recommendations [6].

TOE to assess LAA complete occlusion and exclude thrombosis was routinely performed three months after the intervention, and a follow-up visit was performed after 12 months.

### 2.4. Statistical Analysis

Continuous variables that were normally distributed were expressed as means ± standard deviation (SD). Continuous variables with non-normal distribution were expressed as medians and interquartile ranges. The Kolmogorov–Smirnov test was used for normal distribution.

Category variables were expressed with numbers and percentage (%).

Kaplan–Meier curves were used for survival analysis for adverse events and analyzed with the Mantel–Cox test.

All statistical analyses were performed using SPSS 24.0 (IBM, Armonk, NY, USA) software, Graphpad prism 4 (La Jolla, CA, USA), and Microsoft Excel 2021 (MICROSOFT CORPORATION, Redmond, WA, USA).

## 3. Results

**Figure 2:**
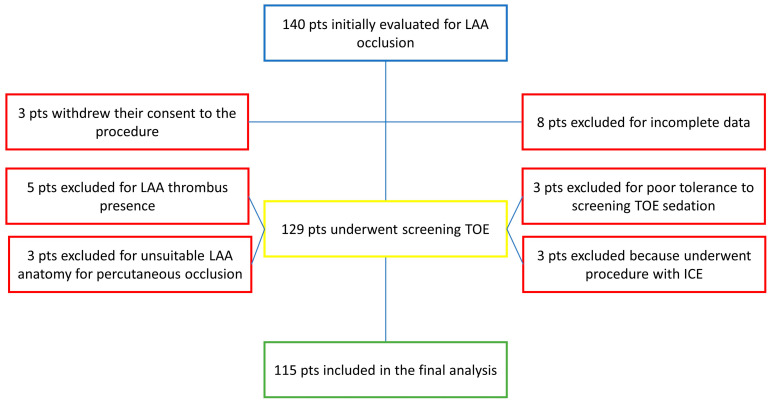
Flow chart of patients included in the analysis.

From 1 October 2018 to 1 October 2022, 140 patients were evaluated for percutaneous LAA occlusion. Three patients were judged to be unable to tolerate the sedation during screening TOE; two of them underwent the procedure under GA, and one underwent the procedure with ICE. Three other patients underwent the procedure with ICE due to esophageal varices, patient preference, and training with the method, respectively. After excluding these patients, those with LAA thrombi, those who refused to be subjected to the procedure, those deemed unsuitable, and those with missing data, one-hundred fifteen patients treated with Watchman/FLX (Boston Scientific Corporation, Marlborough, MA, USA) were included in the final analysis (Figure 2). Of these, 29% were female, with a mean age of 76.4 ± 7.6 years. The baseline characteristics are summarized in Table 1. All the included patients suffered non-valvular AF. The mean CHA_2_DS_2_-VASc Score was 4.4 ± 1.4, and the mean HAS-BLED score was 3 ± 1.1. The patients underwent percutaneous LAA occlusion for different reasons: an absolute contraindication for long-term oral anticoagulation (OAC) due to previous bleeding events (63 pts; 54.8%); intolerance of OAC due to an irreversible predisposition to bleeding—e.g., cerebral amyloid angiopathy—(24 pts; 20.9%); concomitant indication for DAPT; and high-bleeding-risk features (22 pts; 19.1%) or stroke while on OAC (6 pts; 5.2%).

Among those with a history of bleeding, sixteen patients (13.9%) suffered intracranial bleeding, thirty-eight patients (33.1%) suffered from gastrointestinal or genitourinary bleeding, and nine patients (7.8%) suffered from recurrent and severe epistaxis. The patients with OAC intolerance presented the following causes for an irreversible predisposition for bleeding: one patient (0.9%) had cerebral amyloid angiopathy, four patients (3.5%) had severe intestinal angiodysplasia, thirteen patients (11.3%) had chronic severe renal failure, and six patients (5.2%) had bone marrow cell dyscrasias. With regard to atrial fibrillation patterns, they were permanent in fifty-seven (49.6%) cases, paroxysmal in thirty-eight cases (33%), and persistent in twenty (17.4%) patients. Before LAA closure, fifty-five patients (47.8%) were treated with NOAC therapy, and twenty-one (18.2%) were treated with VKA.

The planning TOE allowed for the examination of LAA morphology, revealing a clear prevalence of cauliflower shape (57 pts; 49.6%), followed by chicken wing (27 pts; 23.5%), cactus (11 pts; 9.6%), and windsock shapes (13 pts; 11.3%). In seven (6.0%) cases, morphology was not reported.

The main procedural data are summarized in Table 2. Acute procedural success was obtained in one-hundred fourteen (99.1%) patients. No patients required an anesthesiologist’s support for sedation or ventilation, and no cases of respiratory failure requiring manual or mechanical ventilation were observed. The mean midazolam dose required to obtain CS was 5.9 ± 2.1 mg. Thirty-nine patients (33.9%) required an additional administration of Fentanyl with a mean dose of 52.8 ± 19.6 mcg. The procedural and fluoroscopic times were similar among the patients treated with additional Fentanyl and those who only received midazolam.

During the procedure, in five cases, the device was changed due to under or over sizing at the initial evaluation leading to instability or suboptimal sealing-off in the LAA.

We observed seven major complications (five of which were for patients treated with midazolam only, and two were observed for those treated with midazolam plus fentanyl): in one case (0.9%), the procedure failed because of cardiac tamponade with the necessity of urgent anesthesiologist support and cardiac surgery. Despite this, the patient died within 5 days from the procedure. There were two other cases (1.7%) of mild pericardial effusion; these did not require intervention. Major bleeding events requiring transfusions were reported in three cases (2.6%): in two cases, the bleeding was related to the procedure, whereas the third case required transfusion due to severe hematuria. Another patient suffered from a right-half larynx, soft palate, and oropharynx hematoma developed during the procedure, which was treated with 10-day steroid therapy. In one case (0.9%), a pseudoaneurysm occurred in the site of vascular access. Device thrombosis three months after the procedure was reported in one case (0.9%) and treated with intravenous UFH for 48–72 h, with complete resolution observed at the follow-up TOE procedure. No instances of device embolization, air embolization, or periprocedural pneumonia due to aspiration during conscious sedation were observed. The average length of in-hospital stay was 3.1 ± 3.9 days.

Ninety-eight patients (85.2%) received DAPT at discharge, while seventeen received SAPT (14.8%); regardless of the moment of the suspension, one-hundred fourteen patients (99.1%) stopped receiving DAPT within 12 months. Forty-nine patients (42.5%) must continue lifelong SAPT.

At the 3-month follow-up, three patients died (2.6%), of which two (1.7%) died due to cardiovascular (CV) causes. Four patients (3.5%) suffered from major bleeding events, and three (2.6%) suffered from minor bleeding. One case (0.9%) of pulmonary embolism occurred. The 3-month TOE results showed small leaks (<5 mm) in nine patients (7.8%), while one case of a persistent large leak (≥5 mm) was reported (0.9%).

At a long-term follow-up, after 10 ± 9 months, the following adverse events occurred: 22 deaths (19.1%), seven CV deaths (6%), one stroke (0.9%), one TIA (0.9%), and eight major bleeding events (6.9%). Long-term follow-up adverse events are reported in Table 3.

## 4. Discussion

In recent years, LAA occlusion has gained more visibility and dissemination in order to reduce stroke risk in patients with AF and ineligible for long-term oral anticoagulation. Procedural imaging is crucial to achieve good intervention results. TOE is still the most widely used form of imaging guidance during procedures [11], generally used in combination with fluoroscopy; the need to ease the discomfort of the patient, often with GA, represents its main side effect, resulting in increased costs, a longer duration of the procedure, higher risk, and longer hospitalization [12].

CS is emerging as an alternative to GA in interventional cardiology, such as percutaneous mitral valve repair and transcatheter aortic valve implantation, presenting good results [13,14,15]. Imaging with TOE during the procedure under CS has been the standard of care in our centers for a few years now, with good results in terms of patients’ satisfaction, low cost, and no requirement for arranging cardiac computed tomography angiography (CCTA) or an anesthesiologist’s presence, resulting in a cardiologist-only planning of the procedure.

In the setting of LAA occlusion, despite a general recommendation for performing intervention under GA, CS is widely used in real practice, as confirmed in a 2013 survey where 50% of procedures were performed under CS [16]; however, data about the drugs employed and their safety and planning (e.g., the presence or absence of an anesthesiologist in the cath-lab) are scarce and often very different from center to center.

Most of the papers published on the topic of CS in LAA closure report the use of propofol as the main sedative agent, with midazolam and opiates only used preoperatively [9,17,18,19]. A propofol-free protocol with midazolam and fentanyl has been reported in a few cases [20,21]. Midazolam used in association with dexmedetomidine showed good results in one large study [22]. The results of our study are in line with previous work using midazolam-fentanyl and midazolam-dexmedetomidine protocols, showing good safety with the absence of conversion from CS to GA, while in the propofol protocols, some conversion from CS to GA occurred [9]; the added value of our study is that it reports the results obtained for an unselected population (including patients with high-risk intubation) and is the only one that reports the rate of the feasibility of a procedure under CS at the time of the screening TOE procedure. Moreover, it should be noted that while propofol can provide better results in terms of immobility and deeper sedation, it should be always used by physicians trained in intensive care and with skills in advanced airway management. Midazolam can also be used by an echocardiographer not skilled in airway management, as it was in our case, without the presence of an anesthesiologist on site thanks to its low respiratory depression effect and the availability of a rapid reversal agent. Furthermore, in our case series, there was no need for ventilation in any case, nor an auxiliary manual breathing unit, while this was not specified in previous works, where only the rate of conversion to GA was reported.

Some recent studies evaluated the role of hypnotic communication for analgosedation in patients undergoing subcutaneous implantable cardioverter defibrillator implantation and during transcatheter ablation of atrial fibrillation; in our perspective, future studies should also evaluate the role of hypnotic communication for patients undergoing LAA occlusion in order to limit the use of medications with potential adverse effects [23,24].

Finally, the role of ICE in guiding transcatheter procedures is expanding fast, and in the case of ICE-guided LAA occlusion, CS is already a standard of care. As discussed in the introduction, ICE diffusion is still limited by higher costs and a lack of 3D-imaging tools. In our opinion, becoming an expert in CS in a cath-lab in a more difficult setting like that for TOE-guided procedures, in perspective, will be useful for interventional cardiologists in the future when the diffusion of ICE will be greater.

In our study, a perceived long length of stay might have been reported; this is due to the fact that many patients were subjected to LAA closure during hospitalization for other reasons (e.g., acute coronary syndrome) and because of procedure refund rules in our country. Moreover, a long procedural time might have been observed; the reason for this is that, in our centers, there is always an interventional cardiologist in training during every procedure.

Our study has limitations due to its small sample size and retrospective, non-randomized, and observational design. The low number of ICE-guided procedures did not allow us to perform potentially interesting comparisons like comparing the LAA occlusion procedure with TOE guidance under CS vs. the LAA occlusion procedure with ICE. Because of its retrospective nature, data concerning the satisfaction of the patients regarding sedation are not available; however, the utilization of agents provoking anterograde amnesia (such as midazolam) confers a good memory of the experience during the procedure.

Selection bias might have influenced our study because some patients might not have been recorded in our dataset. Long-term data were collected via a follow-up call or with the help of an administrative database. Our experience concerns Watchman/FLX devices, which have a lower incidence of procedural complications, as shown in the SWISS APERO trial (Comparison of Amulet Versus Watchman/FLX Device in Patients Undergoing Left Atrial Appendage Closure), and, for this reason, is not extendable to other devices [25]. Larger, randomized studies are needed to compare clinical outcomes and patient discomfort between different imaging modalities (TOE vs. ICE) and different anesthesia modalities (GA vs. CS). In particular, as reported by clinicaltrials.gov, many studies are actively recruiting patients to evaluate the best imaging modalities for guiding LAA occlusion procedures. These studies compare the roles of cardiac computed tomography angiography (CCTA), ICE, and LAA radiography in planning the LAA occlusion procedures evaluating morphology, dimensions, and the presence of LAA thrombi before and during the percutaneous intervention. In particular, NCT04524741 is comparing the use of ICE vs. LAA radiography in a 1:1 ratio, evaluating the procedure/fluoroscopic time, contrast agent consumption, radiation dose, peri-procedure complications, occluder device size, and the occlusion success rate. Similar works (NCT05051280, NCT04640051, and NCT04913207) are evaluating the role of CCTA and three-dimensional computed tomography angiography technology (3D-CTA) in guiding the selection of LAA occluder device size. The different imaging approaches to the planning of LAA occlusion procedures in the future can modify the workflow of the patients planned for LAA occlusion and possibly reduce the role of TOE imaging, removing the main source of discomfort for the patients treated with this percutaneous intervention.

## 5. Conclusions

In our study, LAA occlusion with the Watchman/FLX device performed under conscious sedation with a midazolam-fentanyl protocol and without the presence of an anesthesiologist on site was successful, with a low rate of adverse events and no need for ventilation or conversion to GA. Performing this procedure without anesthesiological assistance can be useful in term of cost, side effects, length of procedure, and hospitalization, without incurring a higher risk for the patients, even when TOE is performed for procedural imaging.

## Figures and Tables

**Table 1:** Baseline characteristics of patients who underwent percutaneous LAA occlusion.

Baseline Characteristics	N = 115
Male sex	82 (71%)
Female sex	33 (29%)
Age (years)	76.4 ± 7.6
Dyslipidemia	50 (43.4%)
Smoking (prior or current)	36 (31.3%)
Liver disease	5 (4.3%)
Creatinine	1.7 ± 1.87
Coronary artery disease (CAD)	52 (45.2%)
Prior acute myocardial infarction (AMI)	43 (37.3%)
Prior percutaneous coronary intervention (PCI)	47 (40.8%)
Prior coronary artery bypass graft (CABG)	8 (6.9%)
Pacemaker carriers	10 (8.6%)
Prior stroke	25 (21.7%)
Prior transient ischemic attack (TIA)	6 (5.2%)
Heart failure	29 (25.2%)
Peripheral artery disease (PAD)	12 (10.4%)
Normal ejection fraction	57 (49.5%)
Reduced ejection fraction (<50%)	44 (38.2%)
Paroxysmal AF	38 (33%)
Persistent AF	20 (17.4%)
Permanent AF	57 (49.6%)
CHA_2_DS_2_-VASc Score	4.4 ± 1.4
HAS-BLED	3 ± 1.1

**Table 2:** Procedural characteristics of percutaneous LAA occlusion. * The patient who suffered a cardiac tamponade was the only one requiring urgent anesthesiological support.

Procedural Characteristics	N = 115
LAA occlusion device	
Watchman/FLX	115 (100%)
Device size (mm)	27 ± 4
Device compression (%)	25.2 ± 5.1
Acute procedural success	114 (99.2%)
Procedural time (min)	87.6 ± 21.4
Fluoroscopic time (min)	13.9 ± 8.7
Device size change during procedure	5 (4.3%)
Dose of drugs	
Midazolam (mg)	5.9 ± 2.1
Need for Fentanyl	39 (33.9%)
Fentanyl (mcg)	52.8 ± 19.6
Complications	
Cardiac tamponade *	1 (0.9%)
Mild pericardial effusion/hemopericardium	2 (1.7%)
Major bleeding events requiring transfusions	3 (2.6%)
Vascular access site pseudoaneurysm	1 (0.9%)
Oropharynx/larinx hematoma	1 (0.9%)
Device thrombosis	1 (0.9%)
Periprocedural Pneumonia	0
Periprocedural Stroke or TIA	0
Device embolization	0
Air embolization	0

**Table 3:** Three-month and long-term adverse events. NA: not applicable.

Adverse Events	Within 3 Months	Long-Term
Stroke	0	1 (0.8%)
TIA	0	1 (0.8%)
Major bleeding	4 (3.5%)	8 (6.9%)
Minor bleeding	3 (2.6%)	3 (2.6%)
Device thrombosis	3 (2.6%)	NA
Large leak in device (≥5 mm)	1 (0.8%)	NA
Small leak in device (<5 mm)	9 (7.8%)	NA
Pulmonary embolism	1 (0.8%)	2 (1.7%)
Death	3 (2.6%)	22 (19.1%)
CV death	2 (1.7%)	7 (6%)
Non-cardiovascular death	1 (0.8%)	14 (12.1%)
Sudden death	0	0

## Data Availability

The data presented in this study are available on request from the corresponding author. The data are not publicly available due to privacy reasons.
